# MYC Immunohistochemistry Predicts *MYC* Rearrangements by FISH

**DOI:** 10.3389/fonc.2017.00209

**Published:** 2017-09-21

**Authors:** Julum Nwanze, Momin T. Siddiqui, Keith A. Stevens, Debra Saxe, Cynthia Cohen

**Affiliations:** ^1^Department of Pathology and Laboratory Medicine, Emory University Hospital, Atlanta, GA, United States; ^2^Department of Pathology and Laboratory Medicine, Tulane University Hospital, New Orleans, LA, United States

**Keywords:** *MYC*, immunohistochemistry, Burkitt lymphoma, *in situ* hybridization, *MYC* rearrangement

## Abstract

*MYC* is the proto-oncogene classically associated with Burkitt lymphoma (BL) located at chromosomal locus 8q24. Rearrangements of *MYC* are seen in nearly 100% of BL but have been reported in 3–16% of diffuse large B-cell lymphomas (DLBCLs). Rearrangements of *MYC* are tested for by flourescence *in situ* hybridization (FISH). In this study, we compared immunohistochemistry (IHC) using a monoclonal antibody directed against the human Myc protein to the current method, FISH. 31 cases were identified that had been tested for *MYC* rearrangements by FISH over 27 months with heterogeneity in the diagnoses: 5 BL; 10 DLBCL; 3 B-cell lymphoma unclassifiable between DLBCL and BL; 5 B-cell lymphoma not otherwise specified; 1 EBV-related B-cell lymphoma; 1 composite CLL/SLL-large cell lymphoma; and 6 designated as high-grade or aggressive B-cell lymphoma. Analysis by FISH was performed as part of the clinical workup, where a *MYC* rearrangement is defined as a split fusion signal in at least 5.7% of cells. Myc-IHC was interpreted as a qualitative positive (overexpressed) or negative (not overexpressed) result. 12 cases (39%) were positive for *MYC* rearrangements by FISH. Overall, 13 cases (42%) showed Myc overexpression by IHC, 11 of which harbored a *MYC* rearrangement by FISH. There were two false positives and one false negative. Thus, Myc-IHC predicted a *MYC* rearrangement by FISH with 92% sensitivity and 89% specificity. We can thus conclude that Myc-IHC should be a potentially useful screening tool for identifying lymphomas that may harbor a *MYC* rearrangement.

## Introduction

The *MYC* proto-oncogene encodes a multifunctional, nuclear phosphoprotein that plays a role in cell cycle progression, apoptosis, and cellular transformation (1). *MYC* was first discovered as the cellular homolog of the retroviral V-MYC oncogene identified from studies of oncogenic retroviruses (2, 3). Soon after, chromosomal translocations juxtaposing *MYC* to immunoglobulin enhancers were documented in B-cell Burkitt lymphomas (BLs) (4). These were located at chromosomal locus 8q24 (4). Subsequently, mutations, overexpression, rearrangement, and translocation of this gene have been associated with various hematopoietic tumors, leukemias, and lymphomas (5).

A major effect of *MYC* is B-cell proliferation (6, 7). *MYC* gene alterations have been identified in mature B-cell neoplasms that are usually associated with an aggressive clinical behavior (8). In the United States, mature B-cell neoplasms account for approximately three-quarters of all lymphoid neoplasms (9). They comprise the majority of diffuse large B-cell lymphoma (DLBCL), BL, chronic lymphocytic leukemia/small lymphocytic lymphoma (CLL/SLL), follicular lymphoma, and plasma cell neoplasms (9). B-cell neoplasms are also the fourth most common childhood cancers accounting for about 6% of pediatric malignancies with the most prevalent entities being BL (43%) and diffuse B-cell lymphoma (13%) (10).

C-Myc, N-Myc, and L-Myc are the three members of the Myc oncoprotein family known to play a role in the pathogenesis of numerous human neoplastic diseases (11). C-Myc overexpression is invariably associated with BL (12). Furthermore, rearrangements of the *MYC* gene are seen in nearly 100% of BL with the most common translocation variant being t(8;14) (q24; q32) (8), which accounts for approximately 85% of cases (13). Other less common translocations, such as t(2; 8) (p12; q24) and t(8; 22) (q24; q11), account for the remaining 15% of cases (13). In contrast, DLBCL that includes a heterogeneous group of intermediate to high-grade mature B-cell neoplasms is reported to have rearrangements of *MYC* in 3–16% of cases (14).

Myc overexpression that results from dysregulation in the cell cycle of the Myc protein can be assayed by Western blot or immunohistochemistry (IHC) (15). There is increasing evidence that Myc overexpression has a prognostic importance that may trump cytogenetic findings. There is, as yet, no definitive evidence of a role as a predictive biomarker (15).

Rearrangements of *MYC* are typically tested for by fluorescence *in situ* hybridization (FISH). This method is currently considered the most accurate method for detection of oncogene amplification in human tumors (16) and is the gold standard for prediction of *MYC* rearrangement (17). However, the procedure is laborious, demanding, and expensive due to the need for a fluorescence microscope (16).

Immunohistochemistry is less tedious to perform and less expensive than FISH. It has the potential to reduce the number of FISH specimens if specificity was high (15). Recently, a monoclonal antibody became commercially available for IHC, made by Epitomics (Burlingame, CA, USA). It is a rabbit antihuman immunoglobulin G molecule, clone Y69 (18) produced from a synthetic peptide that corresponds to residues in the N-terminus of human C-Myc. The antibody also bears cross-reactivity to mouse and rat C-Myc (18).

The purpose of this study is to evaluate the effectiveness of Myc-IHC in predicting *MYC* rearrangement by FISH (the current gold standard) in mature B-cell lymphomas.

## Materials and Methods

A search was made through the surgical pathology files of Emory University Hospital, Atlanta, GA, USA, for cases that had been tested for *MYC* rearrangements by FISH. A total of 31 cases were identified over 27 months (May 2011–July 2013) with heterogeneity in the diagnoses: 5 BL; 10 DLBCL; 3 B-cell lymphoma unclassifiable between DLBCL and BL; 5 B-cell lymphoma not otherwise specified (NOS); 1 EBV-related B-cell lymphoma; 1 composite CLL/SLL-large cell lymphoma; and 6 designated as high-grade or aggressive B-cell lymphoma. All samples had been obtained in accordance with guidelines approved by the Emory IRB committee.

### Myc IHC

Sections (5 μm) of formalin-fixed, paraffin-embedded tissue are tested for the presence of antigen using the Bond Polymer Refine Detection Kit (DAB chromogen) (Leica Microsystems, Bannockburn, IL, USA). The detection system avoids the use of streptavidin and biotin and, therefore, eliminates non-specific staining as a result of endogenous biotin. All steps are performed on the Leica Bond Maxx III automated system. Specimens are deparaffinized and antigen retrieved (pH 6.0 in citrate buffer for 20 min) on the instrument. All slides are incubated with rabbit monoclonal antibody (Epitomics, clone Y69; 1:100; Abcam, Cambridge, MA, USA) for 15 min, with post-primary polymer for 8 min, blocked with 3% hydrogen peroxide for 5 min, 3,3-diaminobenzidine (DAB, brown chromogen) for 10 min, and hematoxylin as counterstain for 5 min. These incubations were performed at room temperature; between incubations, sections were washed with Tris-buffered saline (Bond wash solution). Coverslipping was performed using the Tissue-Tek SCA (Sakura Finetek USA, Inc., Torrance, CA, USA) coverslipper. Positive controls of known positive tissues (BL) and negative controls with specific antibody replaced with Tris buffer were run with the patient/study slides.

Myc-IHC was interpreted by the authors, blinded to the FISH result, as a qualitative positive (overexpressed) or negative (not overexpressed) result. A positive result is represented by a strong Myc nuclear staining in greater than 50% of the tumor cells. A negative result is represented by a usually faint staining in a small percentage of cells (less than 50%).

### Fluorescence *In Situ* Hybridization for MYC Gene Rearrangements

FISH performed at Emory University was carried out using formalin-fixed, paraffin-embedded tissues, with 4 μm sections mounted on positively charged slides. Slides were deparaffinated on a slide warmer for 2 h. The slides were processed on a VP 2000 (Abbott Molecular, Abbott Park, IL, USA), which includes pretreatment with protease. After slides were processed, 5 μl of *MYC* break apart probe (Abbott Molecular, Inc.) was added, and the slides were coverslipped and sealed with rubber cement. The slides were then placed in a hybridization chamber overnight at 37°C. Slides were washed with 2× SSC/0.3 NP-40 and dehydrated with EtOH. Slides are counterstained with 10 μl DAPI and chilled in the freezer for a minimum of 20 min. A total of 200 cells were analyzed by two different readers each reading 100 cells. The specimen was considered positive for a MYC rearrangement if >6% of the cells demonstrate a single fusion and a separate green and red signal.

## Results

**Figure 1:**
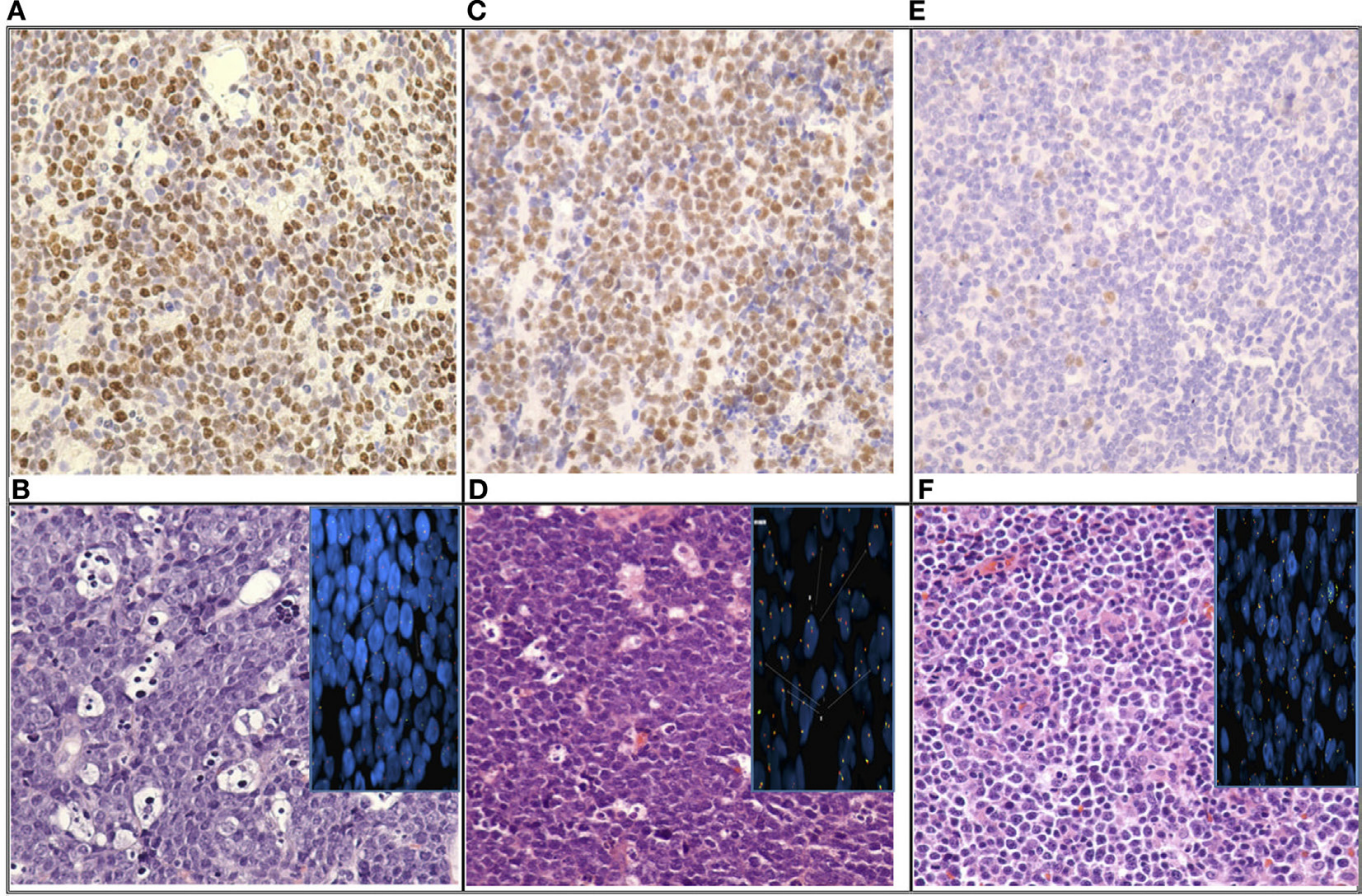
Micrographs of immunohistochemistry and FISH slides; **(A)** “True positive”: Myc nuclear staining in nearly 100% of tumor cells; **(B)** “True positive”: Burkitt lymphoma (BL) (H/E) with *MYC* rearrangement by FISH (inset); **(C)** “False positive”: Myc nuclear staining in nearly 100% of tumor cells; **(D)** “False positive” B-cell lymphoma unclassifiable with features intermediate between diffuse large B-cell lymphoma (DLBCL) and BL (H/E) without MYC rearrange by FISH (inset); **(E)** “True negative”: Myc nuclear staining faint in a small percentage of cells; **(F)** “True negative”: DLBCL (H/E) without *MYC* rearrangement by FISH (inset) (image magnification: 4×).

**Figure 2:**
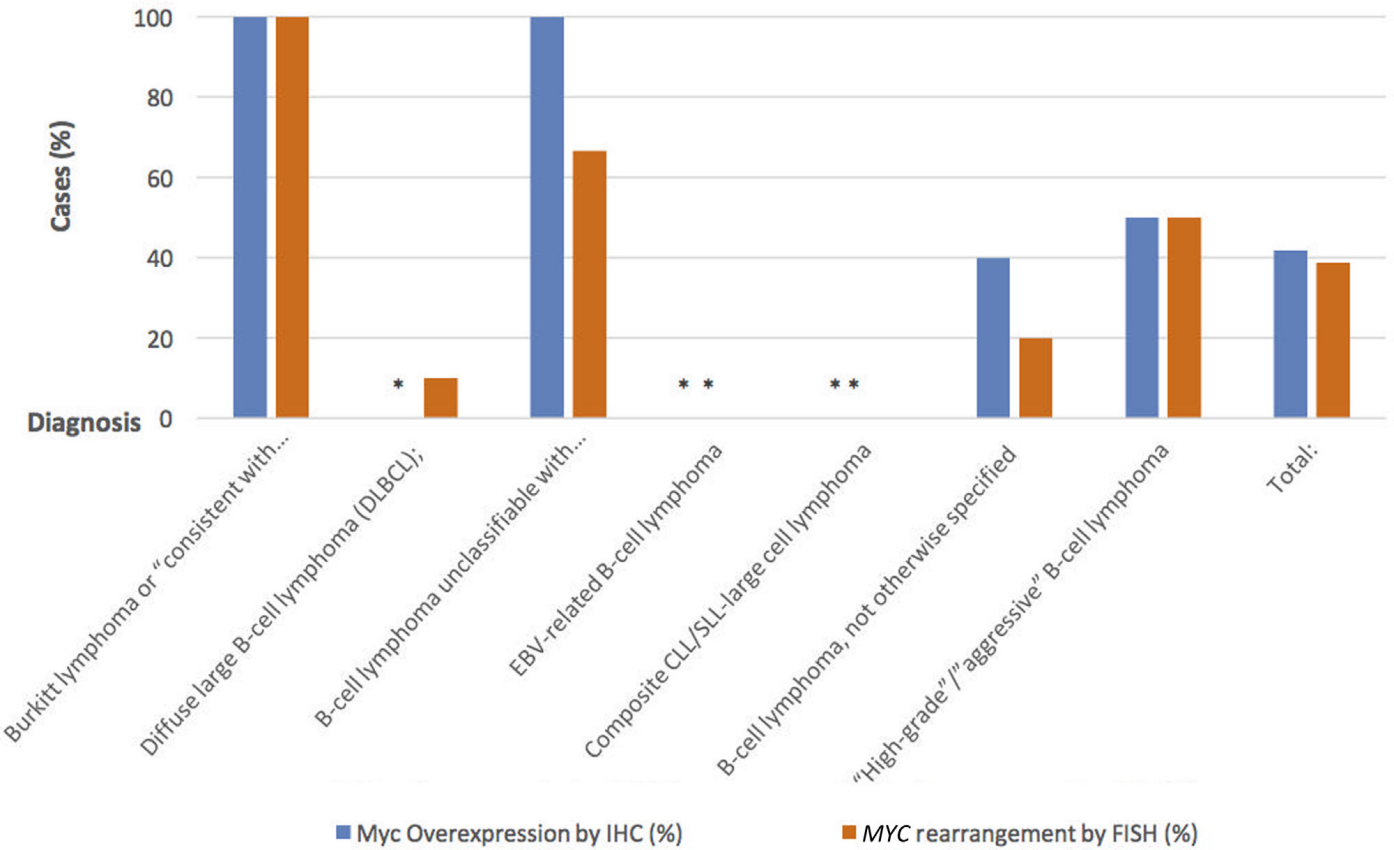
Percentage of positive cases using Myc-IHC compared to positive cases using *MYC* rearrangement by FISH.

Table 1 is a summary of the results of Myc-IHC and FISH by diagnosis. Figure 1 shows micrographs of IHC and FISH slides. 13/31 cases showed overexpression of Myc by IHC (Figure 1A). This is in contrast to 12/31 (38.7%) cases that were positive for *MYC* rearrangements by FISH with the break-apart probe (Figure 1B). One case of B-cell lymphoma unclassifiable with features intermediate between DLBCL and BL and one case of B-cell lymphoma NOS showed Myc overexpression by IHC. Both cases, however, failed to show a rearrangement by FISH and were thus regarded as IHC false positives (Figure 1E,F). The presence of polysomy of chromosome 8 was noted. One case of DLBCL that showed a *MYC* rearrangement by FISH was negative by IHC, which was regarded as a false negative. Figure 2 shows the percentages of positive cases for each test.

**Table 1:** Summary of results by diagnosis.

Diagnosis	Number (%)	Myc overexpression by immunohistochemistry (%)	*MYC* rearrangement by FISH (%)
Burkitt lymphoma (BL) or “consistent with BL”	5 (100)	5 (100)	5 (100)
Diffuse large B-cell lymphoma (DLBCL)	10 (100)	0 (0)	1 (10)
B-cell lymphoma unclassifiable with features intermediate between DLBCL and BL	3 (100)	3 (100)	2 (66.67)
EBV-related B-cell lymphoma	1 (100)	0 (0)	0 (0)
Composite CLL/SLL-large cell lymphoma	1 (100)	0 (0)	0 (0)
B-cell lymphoma, not otherwise specified	5 (100)	2 (40)	1 (20)
“High-grade”/“aggressive” B-cell lymphoma	6 (100)	3 (50)	3 (50)
**Total**	**31 (100)**	**13 (41.9)**	**12 (38.7)**

Table 2 gives results of the statistical analysis. When compared with *MYC* gene arrangements by FISH, Myc-IHC has a sensitivity of 91.6% and a specificity of 89.4%. The positive predictive value in this group of morphologically aggressive-appearing lymphomas was 84.6%, and the negative predictive value was 94.4%. Chi-squared analysis also demonstrated a strong correlation between *MYC* gene arrangements and Myc-IHC (*p* < 0.005). Kappa analysis between both tests gave a score of 0.80.

**Table 2:** Sensitivity and specificity of Myc-IHC compared to *MYC* rearrangement by FISH.

		*MYC* rearrangement by FISH		Total	
		Positive	Negative		
Myc-IHC	Positive	11	2	13	PPV—84.6%
	Negative	1	17	18	NPV—94.4%
	**Total**	**12**	**19**	**31**	
		Sensitivity—91.6%	Specificity—89.4%		

## Discussion

*MYC* is a significant oncogene, and its deregulation has been shown to lead to the development of aggressive lymphomas (19). In the last few years, the importance of *MYC* deregulation has become more apparent; it has been shown to be present in virtually all cases of BL (12, 20), and although present in a minority of DLBCL (3–16%) (12, 14), its presence in DLBCL is associated with poor response to treatment and a poor overall prognosis (21–23). This has made the detection of *MYC* translocation critical in the management of certain cases of DLBCL (24).

While Myc expression may be detected by IHC, *MYC* gene rearrangements are detected by other molecular techniques such as FISH. IHC is a routine method in most pathology laboratories; however, IHC analysis is based on a subjective interpretation of staining intensity (25). IHC is also prone to poor tissue fixation, and there are some issues with reproducibility (26). Currently, FISH is the most sensitive and specific method for detection of oncogene amplification in human tissue samples and it is therefore seen as the gold standard method (26). The main advantages of FISH are its high sensitivity and specificity (16). Also, it is possible to analyze archival formalin-fixed tumor samples as DNA is less subject to effects of tissue fixation and processing than protein (26). In addition, internal controls can be included in each assay and results are quantitative (26). Nevertheless, the FISH method is laborious and demanding, which makes it time-consuming, highly trained personnel are required, and a fluorescence microscope is needed, which makes it a relatively expensive procedure. It is also difficult to study the detailed morphological features of a tumor as FISH signals fade over time (26).

Previous studies have demonstrated that Myc protein expression correlated with gene status in BL and DLBCL (24, 27, 28). Tapia et al. also showed that overall, *MYC* translocated lymphomas had Myc nuclear positivity in 70% of neoplastic cells; in contrast to *MYC* non-translocated lymphomas that were positive in only 28% of the cells (27). This is consistent with the results of our study, which yielded a high sensitivity and specificity of Myc-IHC; 91.6 and 89.4%, respectively. This is also in keeping with the studies of Lynnhtun et al. who demonstrated Myc-IHC sensitivity and specificity indices of 89 and 88% (at IHC positivity thresholds of 80% or more) (24). Kappa testing of our study showed the degree of agreement between both tests is 0.80. This can be interpreted as a substantial agreement and satisfies the minimum inter rater agreement recommended by most authorities for laboratory tests (29).

Among our cases were two false positives (one case of B-cell lymphomas NOS and one case of B-cell lymphoma unclassifiable with features intermediate between DLBCL and BL), which were also positive for polysomy of chromosome 8. This could mean there is a dosage effect of the *MYC* gene as a reason for Myc overexpression as previously suggested by Gill et al. (30). However, this relationship is yet to be established in lymphomas.

We can, therefore, conclude that Myc-IHC predicts *MYC* rearrangements by FISH (the gold standard) with high sensitivity and specificity. We encountered no problems in the interpretation of My-IHC in this cohort of cases. Despite our findings, it should be noted that our data were gotten from a small series. Additional studies on larger series could be appropriate to validate our findings. Nevertheless, we can postulate that Myc-IHC should be a potentially useful screening tool for identifying lymphomas that may harbor a *MYC* rearrangement as it is more widely available than FISH. It is also easier and quicker than FISH, thus offering potential cost and time savings. Furthermore, Myc-IHC may be of great usefulness in low resource centers that lack access to fluorescence microscopy.

## Author Contributions

JN participated in the research and also helped with the statistical analysis done. He was the principal author of the manuscript. MS helped plan and participated in the research and contributed to the review of the manuscript. KS helped plan and participated in the research. DS helped plan and carry out the research. CC helped plan the experiment and also contributed to the review of the manuscript.

## Conflict of Interest Statement

The authors declare that the research was conducted in the absence of any commercial or financial relationships that could be construed as a potential conflict of interest.
